# Incomplete Screw Thread Engagement of Proximal Fragment: A Possible Failure Risk After Internal Fixation for Femoral Neck Fractures

**DOI:** 10.7759/cureus.41349

**Published:** 2023-07-04

**Authors:** Atsuki Tanaka, Takafumi Hiranaka, Takaaki Fujishiro, Motoki Koide, Yoshihito Suda, Akira Saito, Akihiko Arimoto, Koji Okamoto

**Affiliations:** 1 Department of Orthopaedic Surgery and Joint Surgery Centre, Takatsuki General Hospital, Takatsuki, JPN

**Keywords:** incomplete thread engagement, fixation failure, sliding mechanism, thread length, internal fixation, femoral neck fracture

## Abstract

Background

For successful internal fixation for femoral neck fracture, the sliding mechanism of the screw is important because it can induce inter-fragmental compression. The thread should penetrate the fracture line and be located within the proximal fragment. If screw thread engagement is incomplete and a part of the thread remains within the distal fragment, the screw sliding can be disturbed, potentially leading to fixation failure. We hypothesized that screw thread in the fracture is a risk of fixation failure.

Methods

We studied 133 hips that underwent internal fixation for femoral neck fracture using dual sliding and compression screws (DSCS) with 20 mm threads. The existence of incomplete thread engagement and fixation failure (cut out, perforation, pseudoarthrosis, or femoral neck shortening) were evaluated on anteroposterior hip radiography postoperatively. The distances from the thread end to the fracture line, screw head to the femoral head cortex, and femoral head diameter were measured to analyze their relationships with any incomplete thread engagement and fixation failure. Differences in evaluation data were assessed using Fisher’s exact test, Student’s t-test, and receiver operating characteristic (ROC) analysis.

Results

Forty-six cases had at least one screw with incomplete thread engagement, and the other 87 hips had a complete engagement. The failure rate in the group of hips with incomplete thread engagement was significantly higher (7/46, 15.2%) than that in the group of hips with complete thread engagement (3/87, 3.4%) (*P* = 0.032). Incomplete thread engagement was found in 59 out of 266 screws (22.2%), and a femoral head ≤ 43.9 mm in diameter was associated with an increased risk of incomplete thread engagement. Most incomplete thread engagement screws (81.4%) had < 5 mm thread length within the distal fragment.

Conclusion

A partially threaded screw is a significant risk of fixation failure after internal fixation for a femoral neck fracture. The smaller femoral head diameter increases the possibility of incomplete thread engagement. Shortening the thread length by 5 mm may help to avoid incomplete thread engagement.

## Introduction

Femoral neck fractures are common injuries in elderly people [1]. Early operative treatment is considered to be beneficial for maintaining activities of daily living and in preventing early death after a femoral neck fracture [1-3]. Typical treatments are internal fixation, hemiarthroplasty, or total hip arthroplasty. In the last two decades, the number of internal fixations has decreased because arthroplasties have a lower rate of revision, less postoperative pain, and improved functional recovery [4-6].

On the other hand, internal fixation has advantages over arthroplasty, including comparatively minor damage to soft tissue, shorter operation times, and decreased intraoperative bleeding [7]. However, the higher rate of complications is problematic; in addition to osteonecrosis and subtrochanteric fractures, there may be fixation failures, such as cut out, perforation, pseudoarthrosis, or femoral neck shortening [8]. After a problem such as nonunion occurs, there are many methods such as subtrochanteric valgus osteotomy and grafting with the vascularized fibula, but anatomical reduction and a good implant selection are very important in the first surgery to avoid such additional surgical burdens [9]. For successful internal fixation, fixation devices require sufficient fixation ability and a sliding mechanism that enables dynamic compression between the fragments.

Screws with 20 mm or longer threads are widely used to acquire good fixation in osteoporotic bone [10,11]. In our experience, however, such screw threads sometimes incompletely penetrate the fracture line, and a part of the thread can be inserted beyond the fracture line. This “incomplete thread engagement” disturbs effective sliding and compression and, eventually, could cause fixation failure. We hypothesized that, firstly, incomplete thread engagement may be a risk of failure. Secondly, if sufficient fixation can be acquired using a 15 mm thread, it may be possible to reduce the rate of incomplete thread engagement. To prove these hypotheses, we studied the relationship between incomplete thread engagement and fixation failure and estimated the possible effect of shorter thread screws (15 mm).

## Materials and methods

Patient selection

**Figure 1:**
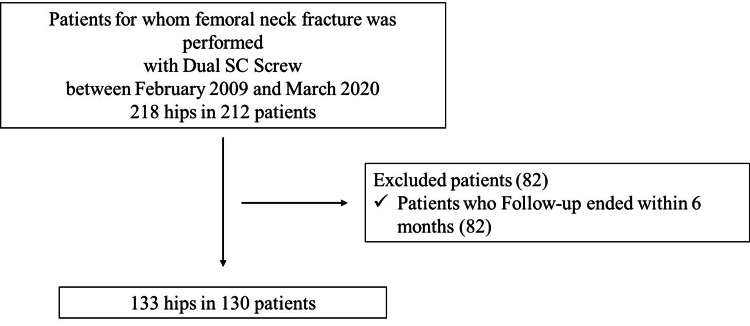
Patient recruitment flow diagram

This retrospective study was performed in accordance with the Declaration of Helsinki and with approval from our institutional review board. The patients provided informed consent for participation. We studied 218 hips in 212 patients that underwent internal fixation for femoral neck fractures using dual sliding and compression screws (DSCS) with 20 mm threads (Teijin Nakashima Medical, Okayama, Japan) between February 2009 and March 2020 (Figure 1). Exclusion criteria were patients who could not be followed up for at least six months and those lacking records of complications. After exclusions, our analysis included 133 hips in 130 patients (105 females and 25 males with an average age of 71.7 ± 13.6 years, and the average BMI was 20.0 ± 3.2, Table 1).

**Table 1:** Patient conditions BMI: body mass index

Demographic	Overall N = 133
Age (years)	71.7 ± 13.6
Sex (male/female)	25/108
BMI (kg/m2)	20.0 ± 3.2
The average observation periods (months)	27.1 ± 23.8
Garden’s stage (I and II/III and IV)	106/27
The data are given as average ± standard deviation	

**Figure 2:**
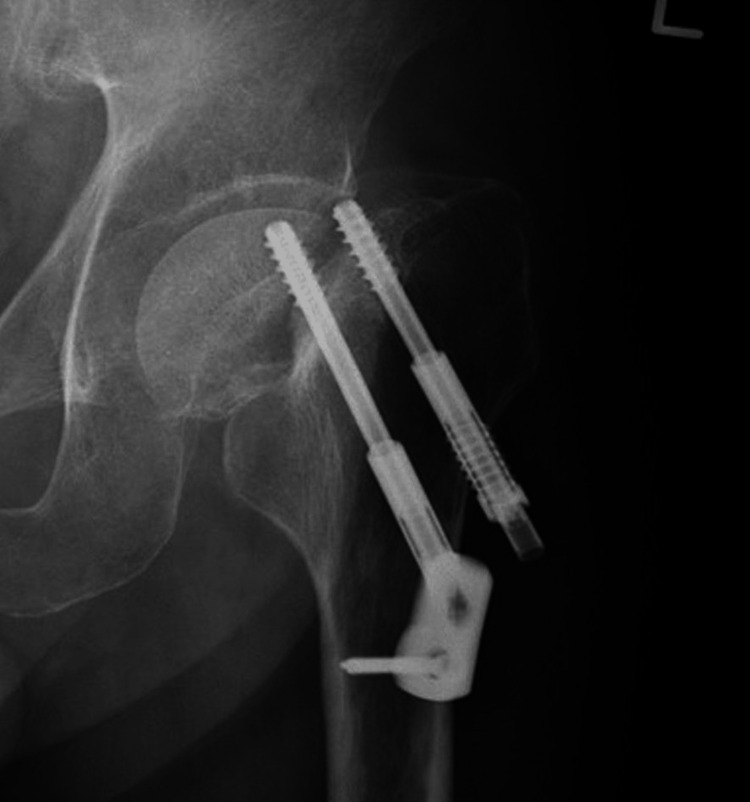
X-ray two years after surgery: fixation failure (cut out) occurred

Follow-up periods of these patients ranged between 6 and 108 months (average observation period: 27.1 ± 23.8 months). Using preoperative radiography, 106 femoral neck fractures were classified as undisplaced fractures: Garden’s stage I and II were 73 and 33, respectively, while 27 femoral neck fractures were classified as displaced fractures: Garden’s stage III and IV were 21 and 6, respectively [12]. In displaced fractures, internal fixation was selected for young patients (< 60 years) or high-risk elderly patients. Failure cases were defined as those with fixation failures, such as cut-out, perforation, pseudoarthrosis, or femoral neck shortening, which required arthroplasty (Figure 2). Failures due to femoral neck necrosis and subtrochanteric fracture were excluded, despite revision surgery being needed.

DSCS

**Figure 3:**
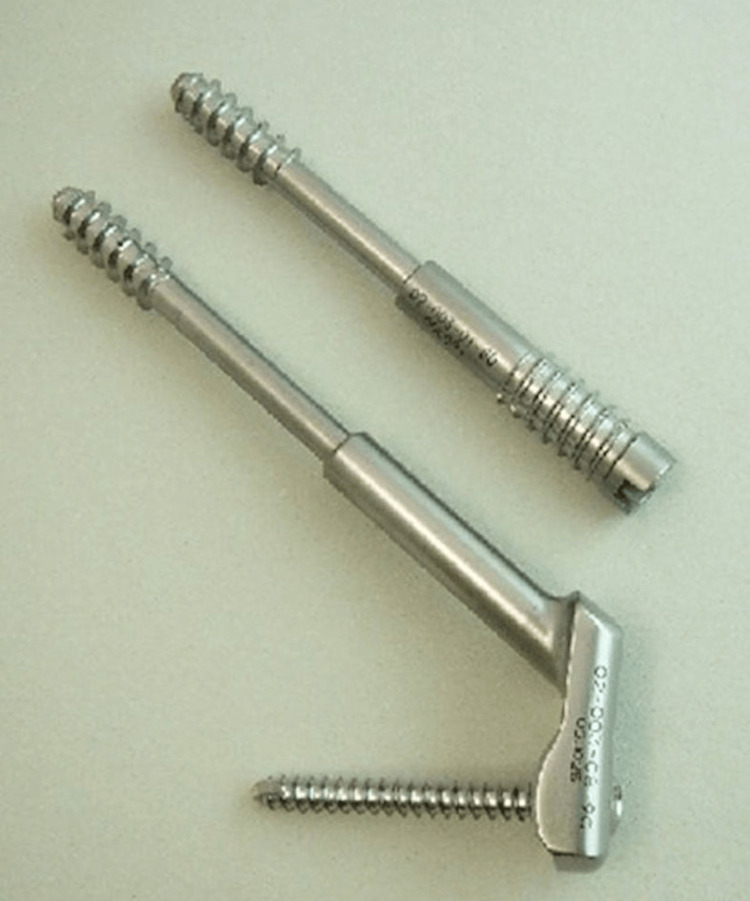
The DSCS with 20 mm threads. It has a screw and a barrel; the screw can move within the barrel. A plate barrel has a one-hole plate with an angle of 135°

DSCS is an implant for femoral neck fractures, comprising a screw and barrel. The screw can slide within the barrel, which induces compression of the fragments. Two types of barrels are available: the screw barrel and the plate barrel. Although both are securely fixed to the lateral cortex of the femur, we routinely used the plate barrel screw to obtain strong angular stability, which has a 135° angle one-hole side plate, along with one thread barrel screw to prevent rotation. In both screws, the thread is 20 mm in length and the shaft diameter is 5.0 mm (Figure 3) [8,13].

Clinical course

Surgery was performed as soon as possible after a diagnosis of a femoral neck fracture. Appropriate traction was applied before the operation. One plate barrel screw and one thread barrel screw were inserted in all cases. The distal screw was inserted just above the calcar femorale, and the proximal screw was inserted at a distance of 16 mm from the distal screw in males and 14 mm in females proximally and slightly posteriorly.

Physical therapy was conducted from the day after the operation, depending on the patients' condition. Immediate full weight bearing was encouraged as much as possible for all patients except for younger patients with displaced fractures. These patients were instead recommended to start partial weight bearing at 6 weeks after the operation.

Radiographical measurements

**Figure 4:**
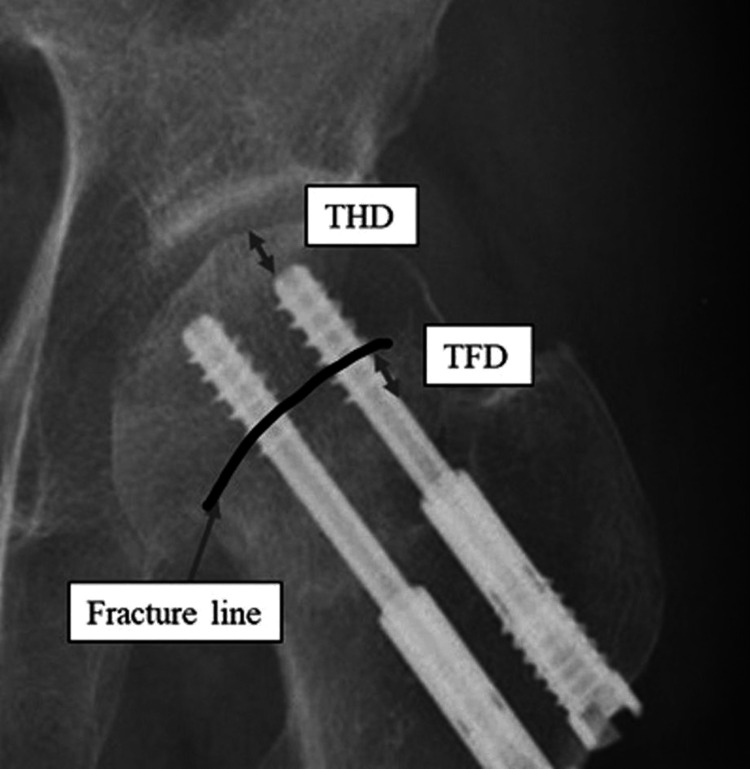
Postoperative X-ray: the TFD and THD were measured. Arrow shows the fracture line TFD: thread-fracture line distance, THD: thread-head cortex distance

**Figure 5:**
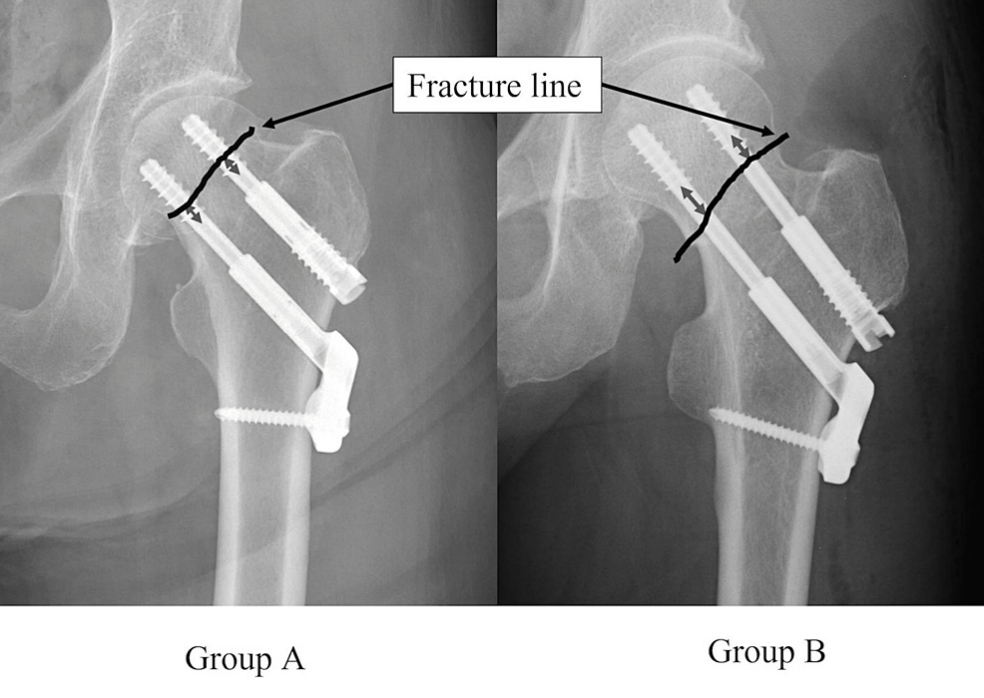
Postoperative X-ray: group A was defined as hips in which at least one screw was beyond the fracture line. Group B was defined as cases in which neither screw was beyond the fracture line

The relationship between screw threads and fracture lines was evaluated on anteroposterior hip radiography within one-week post-surgery. Each screw was divided into “complete” and “incomplete” based on whether the thread completely penetrated the fracture line. The distance from the thread end to the fracture line (thread-fracture line distance (TFD)) was measured (Figure 4). If screw threading was incomplete and a part of the thread was beyond the fracture line, the TFD was expressed as a minus value. The distance between the screw tip and the femoral head cortex (thread-head cortex distance (THD)) was also measured (Figure 4). All hips were classified into one of two groups: "incomplete hips" were those with at least one incomplete thread screw, while "complete hips" were those that comprised two completely threaded screws (Figure 5). The diameter of the femoral head was measured.

Statistical analysis

All measurements are described as average ± standard deviation. Fisher’s exact test was used to determine if there was a significant difference in whether screws were classified as having incomplete thread engagement or not within several groups, such as between the distal and proximal screws, undisplaced and displaced fractures, and males and females. Moreover, the difference in the rate of failure between Group A (incomplete thread engagement) and Group B (complete thread engagement) was assessed using Fisher’s exact test. We also estimated the effect of shortening the thread by 5 mm.

Student’s t-test was used to investigate whether there was a significant difference in the lengths of TFDs between proximal and distal screws and THDs between them. It was also used to analyze significant differences in femoral head diameter between males and females.

ROC curve analysis was used to investigate the relationship between incomplete thread engagement and the diameter of the femoral head. The maximum value of Youden’s index (sensitivity + specificity - 1) was used as the cut-off value. All statistical analyses were performed using easy R (EZR, Jichi Medical University, Saitama, Japan) running on R (R Foundation for Statistical Computing, Vienna, Austria) [14]. P < 0.05 was considered to be statistically significant.

A posthoc power analysis for Fisher’s exact test was performed by G*Power (Heinrich Heine Universitȁt Dȕsseldorf, Germany) using the ratio of fixation failures between groups A and B, using the effect size of 0.33 and α error probability as 0.05. The calculated powers were 0.97, indicating a sufficient sample size [15].

## Results

Screws

The details of the screws are shown in Tables 2 and 3. Of the total of 266 screws, 59 (22.2%) were incomplete thread engagement screws and 207 (77.8%) were complete thread engagement screws. There was no difference in the rate of incomplete thread engagement between proximal and distal screws (26.3% and 18%, P = 0.234) or between displaced and undisplaced fractures (23.6% and 16.7%, P = 0.275). Incomplete thread engagement was significantly more frequent in female than in male hips (P < 0.001).

TFDs were not significantly different between the proximal and distal screws in either complete or incomplete thread engagement (P = 0.166 and 0.140, respectively).

THD was 5.5 ± 2.1 mm for the proximal screw and 7.7 ± 3.5 mm for the distal screw. THD for the distal screw was significantly longer (P < 0.001). Eleven screws had incomplete thread engagement of > 5 mm, all of which were in female patients (Table 3).

**Table 2:** Relationship between screw and fracture line and classification TFD: thread-fracture line distance, THD: thread-head cortex distance

	Overall N = 266		
	Incomplete thread engagement	Complete thread engagement	p-value
Number of screws	59	207	
Number of proximal screws	35	98	0.234
Number of distal screws	24	109	
Number of screws of an undisplaced fracture	50	162	0.275
Number of screws of displaced fracture	9	45	
Number of screws of men	3	47	< 0.001
Number of screws of women	56	160	
TFD of the proximal screw (mm)	-2.9 ± 1.7	5.7 ± 3.6	< 0.001, 0,166
TFD of the distal screw (mm)	-3.7 ± 2.0	5.1 ± 3.8	
THD of the proximal screw (mm)	5.5 ± 2.1	< 0.001	
THD of the distal screw (mm)	7.7 ± 3.5		
The data are given as average ± standard deviation			

**Table 3:** The number of screws divided by TFD. Eleven screws had incomplete thread engagement more than 5 mm TFD: thread-fracture line distance

TFD (mm)	Number of screws (n = 266)
< -5	11
-5--2.5	22
-2.5-0	26
0-2.5	50
2.5-5	67
5 >	90

Hips

**Figure 6:**
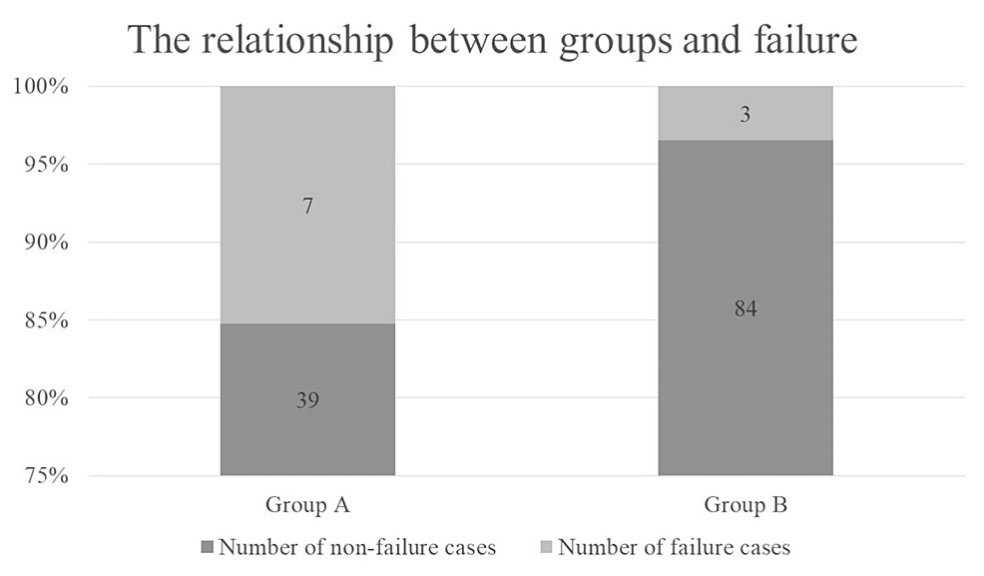
The relationship between incomplete thread engagement and failure. In Group A, seven out of 46 hips (15%) were failure cases. In Group B, three out of 87 hips (3%) were failure cases, with a significant difference between the groups (P= 0.032)

**Figure 7:**
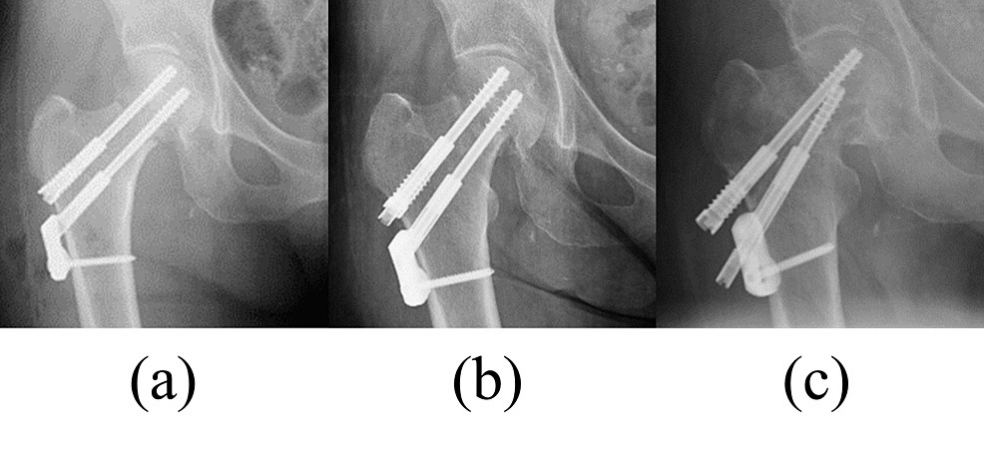
Failure case (a) Postoperative X-ray: a 63-year-old woman underwent internal fixation with a DSCS against femoral neck fracture (Garden’s stage II). In the immediate postoperative period, there was no incomplete thread engagement of either of the screws, it was classified as Group B. (b) X-ray one month after surgery: the thread reached the fracture line. (c) X-ray taken nine months after surgery: due to femoral neck shortening and increased pain, bipolar hemiarthroplasty was performed.

Of the total 133 hips, 46 (34.6%) were classified as incomplete hips and 87 hips (65.4%) as complete hips. In incomplete hips, seven out of 46 hips (15.3%) were cases with failure; three of cutout, three of neck shortening, and one of pseudoarthrosis. In complete hips, three out of 87 hips (3.4%) were cases of failure; one of cutout, one of neck shortening, and one of pseudoarthrosis. There was a significant difference between the groups in failure (P = 0.032) (Figure 6). In all failure cases in the complete hips, the thread reached the fracture line at the time of failure (Figure 7). Comparing only undisplaced fractures, in Group A, six out of 40 hips (15%) were cases with failure, compared with one out of 66 hips (1.5%) in Group B. The rate of failure was thus significantly different between the groups (P = 0.011). Comparing only displaced fractures, in Group A, one out of the six hips (16.7%) was a case of failure, compared with two out of 21 hips (9.5%) in Group B. There was no significant difference between the groups in failure (P = 0.623).

Femoral head diameter

**Figure 8:**
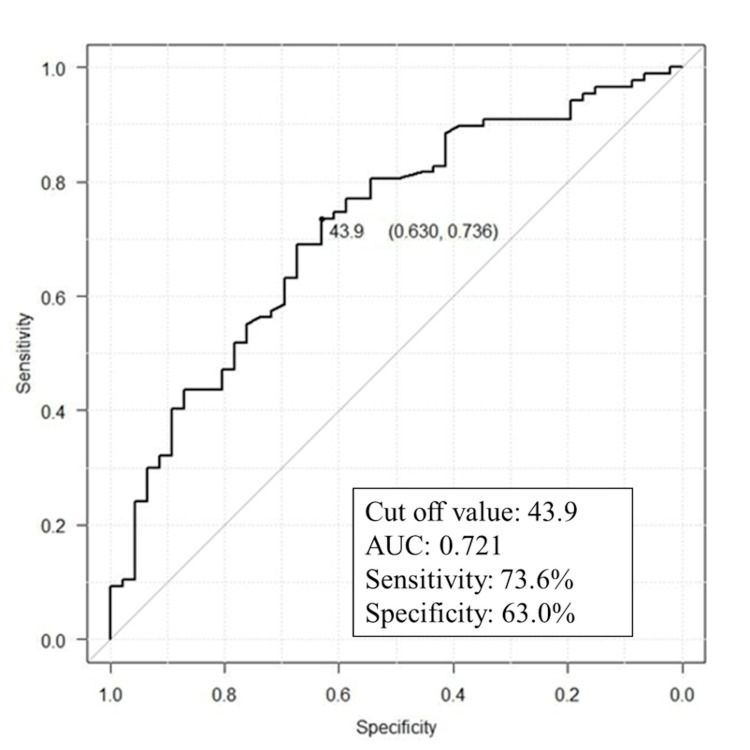
ROC curve analysis was performed to investigate how different diameters of a femoral head affect incomplete thread engagement. The maximum value of Youden's index was used as the cut-off value. The sensitivity, specificity, and AUC were 73.6%, 63.0%, and 0.721, respectively AUC: area under the ROC curve

In male patients, the average diameter of the femoral head was 49.8 ± 3.1 mm. In female cases, it was 44.3 ± 2.6 mm (P < 0.001). ROC curve analysis of the femoral head diameter to predict incomplete thread engagement is shown in Figure 7. The correlation coefficient between TFD and femoral head diameter was 0.384. ROC analysis showed that the cut-off of Youden’s index was a femoral head ≤ 43.9 mm in length with an area under the ROC curve (AUC) of 0.721. Sensitivity and specificity were 73.6% and 63.0%, respectively (Figure 8).

## Discussion

The most important finding of this study was that there was a significantly higher failure rate in incomplete hips compared with complete hips, especially in undisplaced fractures. Moreover, in all cases of failure in Group B (complete thread engagement), where the threads were not beyond the fracture line, at least one thread reached the fracture line before failure occurred (Figure 7). Incomplete thread engagement is thus a significant factor for the occurrence of fixation failure after internal fixation for a femoral neck fracture. Although one report recommends the use of a fully threaded screw as a positioning screw [16], most devices have allowed the sliding of screws to induce the compression of the fragments and accelerate fracture healing [13]. incomplete thread engagement of screws disturbs the sliding mechanism, and it no longer works properly; thus, there is an increase in the risk of failure. To the best of our knowledge, this is the first report to focus on incomplete thread engagement and sliding failure.

In our cohort, incomplete thread engagement was found in 22.1% of screws; it was significantly more common in female hips than in male hips. The diameter of the femoral head of females was significantly smaller than that of males. ROC curve analysis also showed the diameter of the femoral head to be a predictive factor of the occurrence of incomplete thread engagement. According to Sprague et al., the female sex is a risk factor for revision surgery following internal fixation of femoral neck fracture [17]. This may be due to the smaller femoral head diameter in females, which increases the probability of incomplete thread engagement.

Interestingly, particularly in undisplaced fractures, 73 cases (54.9%) were valgus-impacted fractures (Garden’s stage I). Inside a femoral head, the capable distance for inserting screw threads without crossing fracture lines, therefore, becomes shorter, and the number with incomplete thread engagements is increasing. Femoral head diameter and the type of fracture are, therefore, of greater importance in avoiding incomplete thread engagement.

It is notable that most cases of incomplete thread engagement were within 5 mm in distance. The length of incomplete thread engagement < 5 mm accounts for 81.4% of overall cases (Table 3). Failure may be avoided if incomplete thread engagement becomes complete thread engagement. According to Nakanishi et al., the average length of the femoral head depth in Japanese patients is 29 mm [17]. Inferior and superior lines, which insert the screw parallel to the longer femoral neck axis, are, therefore, approximately 25 mm because of the round shape of the femoral head [18]. In cases where the femoral neck fracture occurs close to the sub-capital line, there is only about a 5 mm margin considering that the thread is 20 mm. Moreover, even if it is “undisplaced,” the margin may be even less in the event that ectropion, for example, has occurred. If the thread had been 5 mm shorter, 96% of screws would have avoided incomplete thread engagement, and there may have been increased bone healing. Furthermore, as mentioned, the distance between the thread end and the fracture line should be as far apart as possible to avoid incomplete thread engagement considering the allowance of the sliding mechanism. Shorter threads are preferable for this reason. In short, 15 mm thread screws may be beneficial, as long as the fixation ability of the femoral head is warranted [19].

There are several limitations in relation to this study. First, a large number of patients (n = 82) could not be followed up for more than six months. Many of these patients were transferred to other facilities after surgery and did not visit our hospital after discharge; thus, there may be a greater number of complications than identified. Second, a radiographic evaluation was conducted with X-rays in all cases. Although the screw diameter was used for adjustment, small measurement errors may have been generated and measurement by CT may have been more accurate. Third, DSCS was used as an internal fixation device in all cases. In addition to the DSCS, implants with a sliding mechanism have also been developed, such as Targon FN (B-Braun AG, Melsungen, Germany). Fixation failure in these implants may be a reason for the sliding mechanism not working properly, but a similar analysis should be conducted for other devices.

## Conclusions

Incomplete thread engagement was shown to be a significant risk factor for fixation failure after internal fixation for a femoral neck fracture. A smaller femoral head diameter increases the possibility of incomplete thread engagement. A 15 mm thread decreases it and might contribute to successful internal fixation.
